# *Apt* (Adenine Phosphoribosyltransferase) Mutation in Laboratory-Selected Vancomycin-Intermediate *Staphylococcus aureus*

**DOI:** 10.3390/antibiotics10050583

**Published:** 2021-05-14

**Authors:** Reena Lamichhane-Khadka, Santosh Dulal, Jesus A. Cuaron, Richard Pfeltz, Sushim Kumar Gupta, Brian J. Wilkinson, John E. Gustafson

**Affiliations:** 1 Department of Biology, New Mexico State University, Las Cruces, NM 88003, USA; rlamichh@saintmarys.edu (R.L.-K.); drsantoshdulal@gmail.com (S.D.); jesusacuaronp@gmail.com (J.A.C.); 2 Department of Biology, Saint Mary’s College, Notre Dame, IN 46556, USA; 3 BD Life Sciences, Microbiology Research and Development, Sparks, MD 21152, USA; pfeltz@earthlink.net; 4 Department of Biochemistry and Molecular Biology, Oklahoma State University, Stillwater, OK 74078, USA; sushim.gupta@okstate.edu; 5 School of Biological Sciences, Illinois State University, Normal, IL 61761, USA; bjwilkin@ilstu.edu

**Keywords:** *Staphylococcus aureus*, vancomycin, VISA, adenine phosphoribosyltransferase, *walRK*

## Abstract

Comparative genomic sequencing of laboratory-derived vancomycin-intermediate *Staphylococcus* *aureus* (VISA) (MM66-3 and MM66-4) revealed unique mutations in both MM66-3 (in *apt* and *ssaA6*), and MM66-4 (in *apt* and *walK*), compared to hetero-VISA parent strain MM66. Transcriptional profiling revealed that both MM66 VISA shared 79 upregulated genes and eight downregulated genes. Of these, 30.4% of the upregulated genes were associated with the cell envelope, whereas 75% of the downregulated genes were associated with virulence. In concordance with mutations and transcriptome alterations, both VISA strains demonstrated reduced autolysis, reduced growth in the presence of salt and reduced virulence factor activity. In addition to mutations in genes linked to cell wall metabolism (*ssaA6* and *walK*), the same mutation in *apt* which encodes adenine phosphoribosyltransferase, was confirmed in both MM66 VISA. Apt plays a role in both adenine metabolism and accumulation and both MM66 VISA grew better than MM66 in the presence of adenine or 2-fluoroadenine indicating a reduction in the accumulation of these growth inhibiting compounds in the VISA strains. MM66 *apt* mutants isolated via 2-fluoroadenine selection also demonstrated reduced susceptibility to the cell wall lytic dye Congo red and vancomycin. Finding that *apt* mutations contribute to reduced vancomycin susceptibility once again suggests a role for altered purine metabolism in a VISA mechanism.

## 1. Introduction

*Staphylococcus aureus* is a notorious human pathogen that is associated with both hospital- and community-acquired infections. Since the emergence of multidrug-resistant methicillin-resistant *S. aureus* (MRSA), treatment of infections caused by these organisms has become challenging [1]. The glycopeptide antibiotic vancomycin remains a clinically proven drug for the treatment of serious MRSA infections [2]. Vancomycin binds to the carboxy terminus of the D-alanyl-D-alanine residues of the lipid II peptidoglycan precursor in the cytoplasmic membrane and prevents peptidoglycan synthesis, disrupting cell wall metabolism and ultimately leading to cell death [2,3].

The increased use of vancomycin, in large part due to the increased incidence of MRSA infections, eventually led to the selection of *S. aureus* strains that demonstrated reduced susceptibility and resistance to vancomycin. Based on vancomycin MICs, *S. aureus* isolates are classified as vancomycin-susceptible *S. aureus* (vancomycin MIC ≤ 2 mg/L), vancomycin-intermediate *S. aureus* (VISA) (MIC ≥ 4 mg/L), and vancomycin-resistant *S. aureus* (VRSA) (MIC ≥ 16 mg/L) [2]. A type of VISA termed hetero-VISA (hVISA) initially demonstrate vancomycin MICs of ≤ 2 mg/L, yet upon exposure to vancomycin produce stable VISA subpopulations [2,3].

VRSA strains carry *van* genes acquired from vancomycin-resistant enterococci which modify the peptidoglycan stem peptides and reduce vancomycin binding thereby conferring high-level vancomycin resistance [2]. The VISA phenotype, however, is unrelated to the *van*-mediated VRSA mechanism; the VISA mechanism is supported by chromosomal mutation(s) that are strain dependent and variable [2,3]. VISA mutations lead to alterations in peptidoglycan metabolism and structure, and increased peptidoglycan thickness is common among VISA [3]. VISA strains can also demonstrate reduced autolysis which is thought to contribute to peptidoglycan thickness [2,3]. The overproduction and accumulation of cell wall material in VISA strains, and thus free D-ala-D-ala binding sites, is hypothesized to sequester vancomycin away from its target at the plasma membrane. VISA strains can also demonstrate enhanced cell wall turnover, peptidoglycan cross-linking, teichoic acid synthesis and attenuated virulence [2,3]. Based on a comparison of VISA strain physiology and mutational analysis, it is surmised that the acquisition of the VISA phenotype can occur via multiple evolutionary trajectories [3].

During the early days of VISA characterization, it was hypothesized that the altered regulation of genes involved with purine biosynthesis played a role in a VISA mechanism [4]. Following on this suggestion, another study could not confirm a link between altered purine biosynthetic gene expression and reduced vancomycin susceptibility [5]. The gene *apt* encodes the purine salvage enzyme adenine phosphoribosyltransferase that catalyzes the conversion of adenine and phosphoribosyl pyrophosphate into AMP [6,7] and also plays a role in the uptake of adenine [8,9].

It was reported previously that an *apt* mutation was present in a laboratory-derived VISA strain derived from an MRSA strain [10], although the impact of *apt* mutation was not fully explored. *S. aureus* can form biofilms which represent bacterial cell communities that are encased within extracellular matrices that adhere to surfaces. In *S. aureus* biofilms, the release of extracellular DNA (eDNA) is critical for the production of these multicellular structures [11]. *S. aureus apt* null mutants displayed reduced cell clumping, biofilm formation and extracellular DNA release [12]. Teichoic acids and lipoteichoic acids are major cell wall and membrane components of Gram-positive bacteria that are polymers of glycerol-phosphate and ribitol-phosphate with glycosyl and D-alanyl ester residues [13]. These cell wall molecules participate in multiple metabolic functions such as cation homeostasis, molecular trafficking, presentation of envelope proteins and regulation of autolysins [13]. It has been reported that cell wall teichoic acids play an important role in protecting *S. aureus* from lysis induced by Congo red and other dyes [14]. A *S. aureus apt* null mutant demonstrated “conspicuous resistance” to the dye congo red [12], which is known to target cell wall integrity [14].

Our laboratory reported on the isolation of VISA strains from a clinical hVISA strain (MM66) via vancomycin selection [15]. In an effort to investigate the VISA mechanism of the MM66 VISA mutants, we performed comparative genomic sequencing (CGS), transcriptional profiling and physiological experimentation to corroborate aspects of our omics investigation on these strains. Furthermore, in order to understand an association of the *apt* mutation with the MM66 VISA mechanism, we characterized MM66 2-fluoroadenine reduced susceptibility (FARS) mutants harboring *apt* mutations. The research completed adds to the literature on VISA mechanisms and the characterization of novel alterations that occur within VISA. We also provide evidence that *apt* mutations can support reduced susceptibility to vancomycin.

## 2. Results and Discussion

### 2.1. Mutations Identified in MM66 VISA Mutants

**Figure 1:**
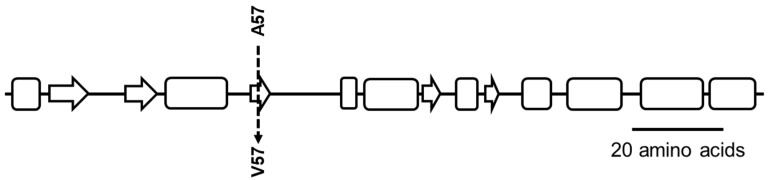
Cartoon indicating the location of the Apt A^57^→V^57^ mutation in the ß3-domain of Apt expressed by MM66-3 and MM66-4. α-helical regions are depicted by rounded rectangles and β-sheet regions are depicted by arrows (secondary structures predicted by Protscale program https://web.expasy.org/protscale/, accessed on 25 January 2021).

CGS data analysis of MM66-3 revealed two intergenic mutations. One was between genes encoding a putative IS1181 transposase and a putative Fur family transcriptional regulator, and another was between two genes encoding hypothetical proteins (SACOL2608 and SACOL2609) (Table 1). CGS also identified six intragenic nonsynonomous mutations in MM66-3. One mutation was located in a putative adenine phosphoribosyltransferase gene *apt*, which leads to an A^57^→ V^57^ (nonpolar A for the larger nonpolar V alteration) within the Apt β3 domain which is part of the five-stranded enzyme core fold that is surrounded by three α-helices [16] (Figure 1). Three additional mutations were located in a single codon of a gene encoding a staphylococcal secretory antigen (*ssaA6*). Six *S. aureus* SsaA homologues [17] including *ssaA6* share a common cysteine, histidine-dependent amidohydrolases/peptidases-amidase (CHAP) domain [18] and amino-terminal signal sequence, suggesting that these proteins play a role in cell wall metabolism [19]. A number of *ssaA* homologues are under the control of *walKR,* which encodes a two-component regulatory system that controls cell wall autolysis in *S. aureus* [20,21]. In MM66-3 the mutations in *ssaA6* lead to a H^297^ → G^297^ (basic H for uncharged G alteration) in the putative peptidoglycan-degrading amidase domain of SsaA6. The fifth mutation was located in a gene encoding a hypothetical protein and the sixth mutation was located within a gene encoding a sulfite reductase (NADPH) flavoprotein alpha-component coding gene *cysJ* (Table 1). All of these sequence discrepancies were confirmed in MM66-3 via PCR amplicon sequencing, yet comparison of all these mutations within the same intergenic regions and genes of 9 *S. aureus* genomes demonstrated that only the *ssaA6* and *apt* were unique to the MM66-3 genome.

We previously reported that CGS analysis identified eight mutations within MM66-4 which included the exact same *apt* mutation found in MM66-3 and a mutation in the histidine kinase *walK* gene [22,23]. The mutation in *walK* leads to a K^263^ → E^263^ (dramatic charge inversion in K to E alteration) in the highly conserved central PAS domain of the sensor module of this sensor histidine kinase [24]. While all eight of these sequence discrepancies were confirmed via PCR amplicon sequencing in this study, nucleotide sequence alignments with nine other *S. aureus* genomes demonstrated that only the *walK* and *apt* mutation are unique to the MM66-4 genome. A reduction in *walRK* activity leads to the reduced transcription of multiple *ssaA* homologues as well as other autolysin genes [19,20] and altered *walRK* transcription and mutations have been implicated in the control of the VISA mechanism [25,26,27,28].

### 2.2. General Aspects of the MM66-3 and MM66-4 Transcriptome

Transcriptional profiling revealed that compared to MM66, MM66-3 demonstrated the upregulation of 97 genes and downregulation of 12 genes (Supplementary Table S1) and MM66-4 demonstrated the upregulation of 143 genes and downregulation of 49 genes compared to MM66 (Table S2). Of these, 79 genes were upregulated and 8 downregulated genes were common to both VISA strains (Table S3). The commonly altered upregulated genes were highly represented by the following functional categories: cell envelope (30.4%), hypothetical/unknown function (16.5%), cellular processes (15.2%), transport and binding proteins (12.7%) and nucleotide metabolism (12.7%) (Table S3). Seventy-five percent of the common downregulated genes were also represented by the cell envelope category (Table S3). An additional 18 genes were upregulated and 4 genes were downregulated in MM66-3 alone, while 64 genes were upregulated and 41 genes were downregulated in MM66-4 alone (Tables S1 and S2). Quantitative real time-PCR (qRT-PCR) confirmed the orientation of transcriptional alteration indicated by microarray analysis in 8 genes (SACOL0660, SACOL1080, SACOL2054, SACOL2182, SACOL0209, SACOL1173 and SACOL2418) (Table 2). 

The *S. aureus lacEFG* encodes the lactose-specific transport components and β-galactosidase [29,30]. The highest upregulated genes in both MM66-4 and MM66-3 were the genes *lacA1*, *lacE* and *lacF* (Table 2). The gene *lacA1* could encode a small (38 amino acid) protein which is transcribed downstream of, and in the same transcriptional direction as *lacR,* the repressor of the *lac* operon [31]. In other VISA strains, *lac* operon genes have been either not transcriptionally altered [4] or are downregulated [32]. The relationship between altered *lac* gene expression and the VISA mechanism is unclear. Compared to MM66, the highest downregulated genes in both MM66 VISA mutants were virulence genes (e.g., SACOL1164, SACOL1173 and SACOL2418) (Table 2 and Table S3).

The alternative sigma factor B (*sigB*) is required by *S. aureus* to respond to stressful environments and antimicrobials [33,34,35,36,37,38]. Besides SigB, the *sigB* operon also encodes RsbU which positively regulates SigB activity, the anti-*sigB* factor RsbW, and RsbV which acts as an anti-anti-SigB factor [35]. Compared to MM66, *sigB*, *rsbW* and *rsbV* were all upregulated in the MM66-4 transcriptome, and qRT-PCR demonstrated that *sigB* was also upregulated in MM66-3 (Table 2).

### 2.3. Altered Cell Wall Metabolism Genes in MM66 VISA

**Figure 2:**
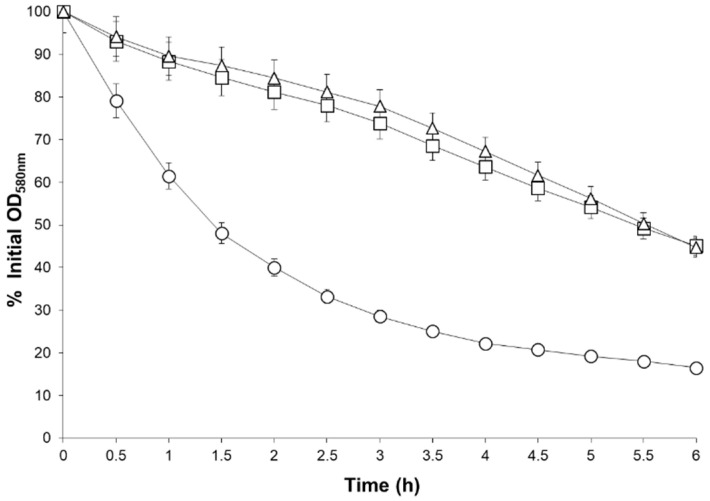
Triton X-100 stimulated whole cell autolysis of parent strain MM66, M66-3 and MM66-4. ○, MM66; ☐, MM66-3; Δ, MM66-4. Error bars represent standard deviation (*n* = 3).

In addition to the *ssaA6* and *walK* mutations, other cell wall metabolism genes demonstrated altered expression in the MM66 VISA mutants. *ssaA1, ssaA2*, and *ssaA3* (Table 2), the autolysin gene *aaa* [39], and *aaa1* (demonstrated 38% identity along its entire length to *aaa*) (Table 2) were all downregulated in MM66-4. In addition, MM66-3 demonstrated reduced expression of the *walRK*-controlled autolysin gene *alt* (Table 2)*. arlS* which encodes the sensor kinase of the two-component regulatory system ArlSR which regulates genes involved with autolysis and cell division [38,40,41,42], was upregulated in both MM66 VISA investigated (Table S3). *arlR* was also upregulated only in MM66-4 (Table S2). ArlRS was initially characterized as a regulator of autolysis [41] and it was reported that ArlRS repressed the expression of the autolysin gene *lytN* [42,43,44]. ArlRS also controls the expression of *atlR* which encodes a gene product that regulates the expression of the autolysin gene *atlA* [42,43]. The gene *murA* which encodes a UDP-N-acetylglucosamine enolpyruvyltranferase that catalyzes the first step of peptidoglycan biosynthesis [45], was upregulated in MM66-4. Lastly, the gene *sceD* which encodes for an autolysin important for cell separation [46] was upregulated in both VISA mutants (Table 2). We hypothesized that akin to other VISA strains [2,3] the MM66 mutants would demonstrate altered whole cell autolysis, and as expected, both MM66 VISA mutants demonstrated reduced whole cell autolysis compared to parent strain MM66 (Figure 2).

### 2.4. Physiological Corroboration of apt Mutation in MM66 VISA Mutants and Characterization of MM66 apt Mutants

**Figure 3:**
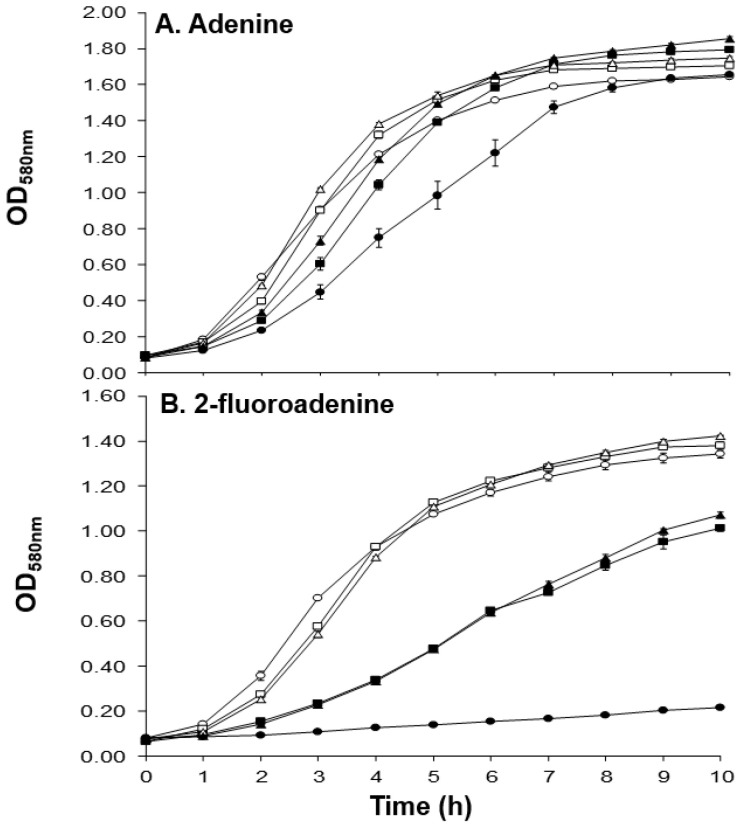
A. Growth of parent strain MM66 and VISA mutants MM66-3 and MM66-4 in the presence of 5 mM adenine. B. Growth of parent strain MM66 and VISA mutants MM66-3 and MM66-4 in the presence of 5 mM 2-fluoroadenine. Open symbols represent control and closed symbols represent growth with 5 mM adenine (**A**) or with 5mM 2-fluoroadenine (**B**). ○, MM66; ☐, MM66-3; and Δ, MM66-4. Error bars represent standard deviation (*n* = 3).

It has been reported that *apt* plays a role in the uptake of exogenous adenine [9,47] and that adenine inhibits the growth of *S. aureus* [48]. Additionally, *apt* mutants of *Bacillus subtilis* exhibited increased tolerance to the toxic adenine analogue 2-fluoroadenine [47]. We therefore hypothesized that the *apt* mutation in the MM66 VISA mutants would reduce adenine and 2-fluoroadenine accumulation and toxicity compared to MM66. As expected, MM66 grew slightly slower than MM66-3 and MM66-4 in the presence of 5 mM adenine (Figure 3) and the growth of MM66 was inhibited by the addition of 2-fluoroadenine, while MM66-3 and MM66-4 grew in the presence of this toxic adenine analogue (Figure 3). Note that MM66 VISA mutants and MM66 exhibited almost identical growth curves in drug-free media (Figure 3). In addition to increased adenine and 2-flouroadenine resistance, we also noted that several genes required for purine (*purLFMNHD*) and pyrimidine (*pyrABFE*) biosynthesis were upregulated in both MM66 VISA mutants compared to MM66 (Table 2).

Selection of *B. subtilis* in 2-flouroadenine resulted in 2-flouroadenine reduced susceptibility (FARS) mutants harboring mutations in *apt* [47]. To ascertain a role for *apt* mutations on vancomycin susceptibility, FARS mutants of MM66 were selected and their *apt* regions were sequenced. Suspected MM66 FARS mutants arose on media containing 5 mM 2-flouroadenine at a mutation frequency of 4.7 × 10^−7^ and all randomly selected MM66 colonies demonstrated higher 2-flouroadenine MICs than MM66 (Table 3). In addition, all FARS MM66 mutants harbored one of a variety of mutations (e.g., deletion, insertion and nonsense mutations) within the *apt* gene (Table 4). The penultimate 2-fluoroadenine MICs could not be determined for all FARS MM66 mutants (Table 3), since 2-flouroadenine precipitated out of the susceptibility testing media at concentrations above 7 mM.

**Figure 4:**
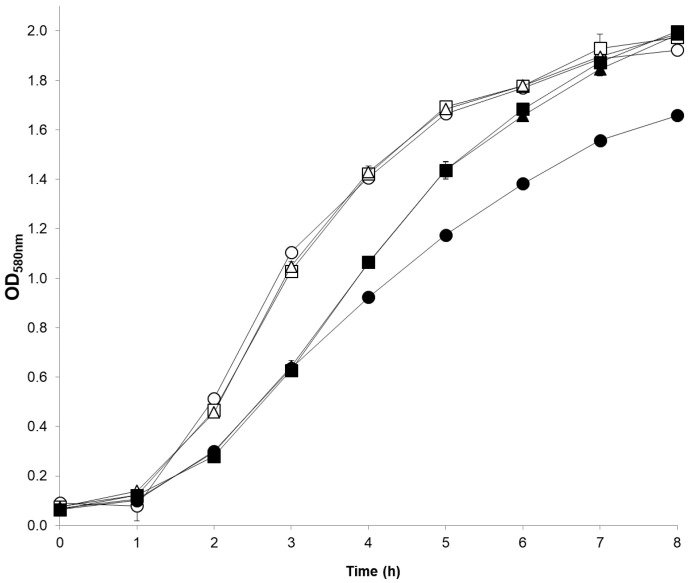
Growth of parent strain MM66 and FARS mutants MM66-FARS-1 and MM66-FARS-6 in the presence of 5 mM adenine. Open symbols represent control and closed symbols represent growth with 5 mM adenine. ○, MM66; ☐, MM66-FARS-1; and Δ, MM66-FARS-6. Error bars represent standard deviation (*n* = 3).

**Figure 5:**
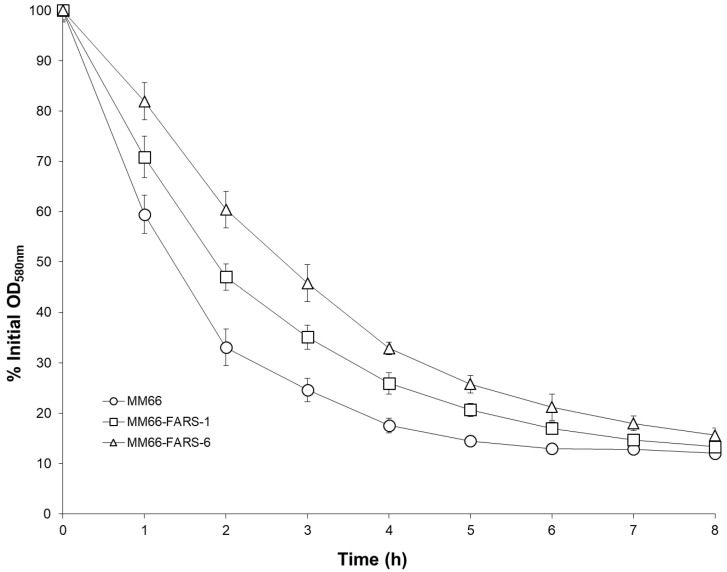
Triton X-100 stimulated whole cell autolysis of parent strain MM66 and FARS mutants. Error bars represent standard deviation (*n* = 3).

**Figure 6:**
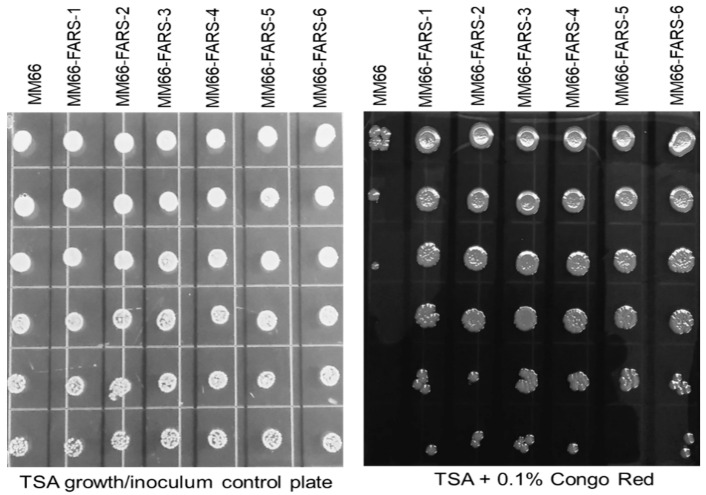
Growth of diluted cultures of MM66 and MM66 FARS mutants on TSA and TSA + 0.1% Congo red.

FARS mutants MM66-FARS-1 and MM66-FARS-6 demonstrated identical growth curves and slightly decreased tolerance to 5 mM adenine growth inhibition compared to the parent strain MM66 (Figure 4). In addition, both MM66-FARS-1 and MM66-FARS-6 demonstrated reduced Triton X-100-stimulated autolysis compared to MM66 (Figure 5). Furthermore, compared to MM66, all MM66-FARS mutants investigated demonstrated increased resistance to Congo red, similar to *apt* mutants previously reported on (Figure 6) [12]. In contradiction to this finding, MM66-3 and MM66-4 demonstrated reduced resistance to Congo red compared to parent strain MM66 (data not shown).

**Figure 7:**
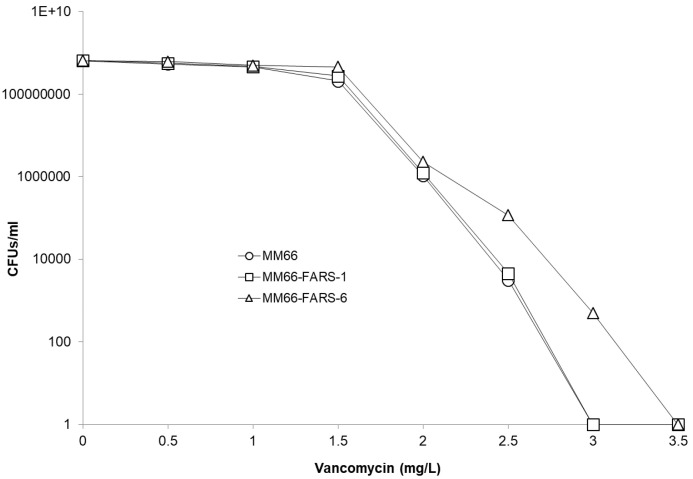
Vancomycin-resistance population analysis of parent strain MM66 and FARS mutants.

Vancomycin resistance population analysis demonstrated that MM66-FARS-6 produced CFUs on 3 mg/L vancomycin while strains MM66 and MM66-FARS-1 stopped producing viable colonies on 2.5 mg/L (Figure 7). However, all FARS MM66 mutants investigated demonstrated increased distances grown on the vancomycin gradients investigated (Table 5).

### 2.5. Alteration in Virulence Factor Gene Expression and Virulence Phenotypes of MM66 VISA Mutants

In a strain-dependent manner, numerous genes encoding virulence factors were either upregulated (e.g., *cap5AB*, *clfA* and *secA*) or downregulated (*coa*, *ssl11*, SACOL1164, SACOL1169, *hly*, *sbi*, *hlgCB*) in MM66-3 and MM66-4 (Table 2). The gene *coa* encodes the virulence factor coagulase which stimulates the clotting reaction of plasma [49] and this gene was downregulated in both MM66-3 and MM66-4 (Table 2). The *hlgACB* operon and *hly* encode hemolysins that lyse erythrocytes [50,51]. *hly* was downregulated in both MM66-3 and MM66-4, and *hlgCB* were both downregulated in MM66-4 (Table 2). In support of these gene expression alterations affecting virulence factor production, MM66-3 and MM66-4 demonstrated increased coagulation time (both VISA mutants = 240 ± 0 min) and reduced hemolytic activity (hemolysis zones = 6 ± 0 mm for MM66-3 and 5 ± 0 mm for MM66-4) compared to MM66 (90 ± 0 min and 10 ± 0 mm hemolysis zone, *p* < 0.005).

### 2.6. Altered Growth of MM66 VISA Mutants in the Presence of Salt

**Figure 8:**
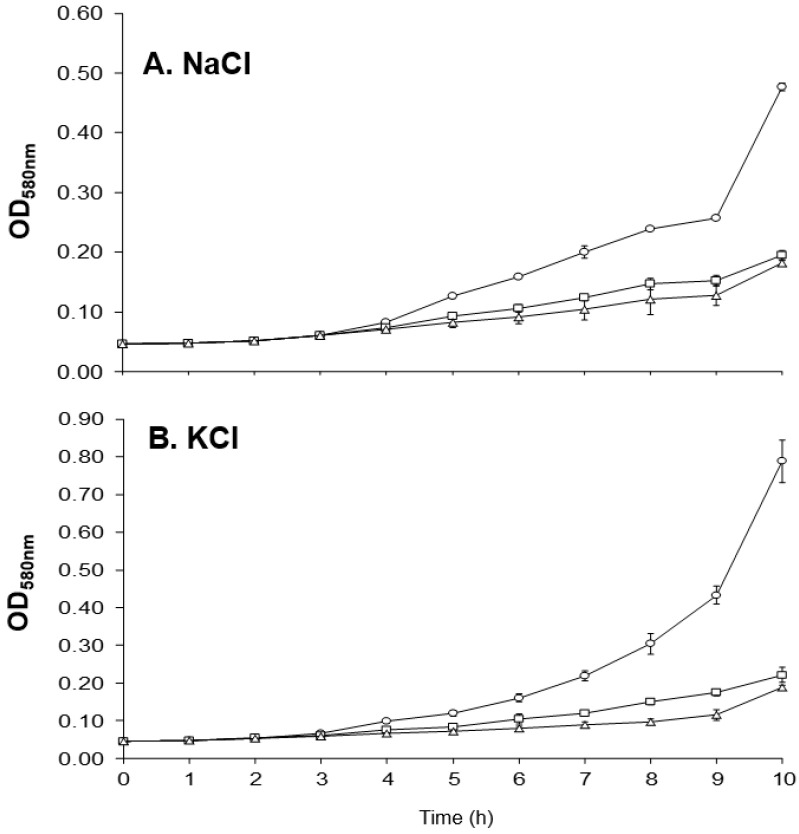
(**A**). Effects of 2 M NaCl on the growth of parent strain MM66 and VISA strains MM66-3 and MM66-4. (**B**). Effects of 2 M KCl on the growth of parent strain MM66 and VISA strains MM66-3 and MM66-4. ○, MM66; ☐, MM66-3; and Δ, MM66-4. Error bars represent standard deviation (*n* = 3).

Osmoprotectants (e.g. choline and glycine betaine) enhance the growth of *S. aureus* at high osmolarity [52] and a variety of gene products are required for the uptake and biosynthesis of these osmoprotectants [53]. The glycine betaine transporter-encoding *opuD1* was upregulated in both MM66-3 and MM66-4 (Table 2). The genes *betA* and *betB*, which encode proteins that catalyze the oxidation of choline to glycine betaine, and *opuD2* which encodes a glycine betaine transporter were downregulated in MM66-4 (Table 2). The qRT-PCR results demonstrated that *betA* was also downregulated in MM66-3 (Table 2). A third gene encoding a glycine betaine transporter component (*opuAC*) that demonstrated 38% identity along its entire length with the OpuAC of *B. subtilis* [54] was upregulated in MM66-4 (Table 2). The data above indicated that the ability of the MM66 VISA to grow in high osmolarity might be altered. In support of this hypothesis, both VISA strains demonstrated reduced growth in the presence of 2 M NaCl and 2 M KCl (Figure 8) compared to parent strain MM66.

## 3. Conclusions

Based on experimental evidence and bioinformatics, we propose that the *ssaA6*, *walK* and *apt* mutations are loss of function mutations. These mutations (*apt* and *ssaA6* in MM66-3, and *apt* and *walK* in MM66-4) in conjunction with the altered regulation of autolysin and cell wall metabolism/envelope genes, contribute to the MM66 VISA mechanism and reduced whole cell autolysis observed. With the exception of the *apt* mutation, this proposal is in line with a number of previous studies that demonstrate the importance of loss of function mutations in VISA mechanisms that directly affect cell wall physiology [3,27,28,55]. Mutations in *walK* lead to the acquisition of the VISA phenotype [25,27,28] and decreased *walRK* expression has been reported to reduce Triton X-100 stimulated whole cell autolysis [19]. While *ssaA6* is a new mutation reported for VISA, it is not difficult to envision a role for a mutated autolysin gene in the MM66-3 mechanism since autolytic activity and autolysin gene expression are commonly reduced in VISA strains [3,27,28,55,56].

Since Apt plays a role in the uptake of adenine [8,9], we propose that the *apt* mutation leads to a reduction in the accumulation of adenine and 2-flouroadenine in the MM66 VISA. This in turn, allowed for the faster growth of MM66 VISA in media amended with adenine or 2-fluoroadenine, compared to MM66. In conjunction with the *apt* mutation, both MM66 VISA also demonstrated increased expression of purine and pyrimidine biosynthetic genes. It is possible that the upregulation of purine biosynthesis genes observed in MM66 VISA is due to defective purine salvage that occurred as a result of the *apt* mutation. Genes involved with purine and pyrimidine biosynthesis have also been reported to be upregulated in VISA with a *walK* or *walR* mutations [25]. It is of interest to note that Apt catalyzes the production and consumption of phosphoribosyl-pyrophosphate, an intermediate metabolite that lies at the intersection between purine and pyrimidine metabolism and carbohydrate metabolism [57,58].

All MM66-FARS mutants that harbored one of a variety of *apt* mutations demonstrated increased 2-flouroadenine MICs. Compared to MM66, MM66-FARS-1 and MM66-FARS-6 also demonstrated identical growth curves, slightly decreased susceptibility to adenine growth inhibition, slightly reduced autolysis and increased resistance to Congo red. Only one FARS mutant demonstrated survival on a higher concentration of vancomycin compared to MM66 in the vancomycin resistance population analysis. Gradient plates utilize a gradual gradient of vancomycin, while population analysis employs abrupt changes in vancomycin concentrations and therefore, gradient plates can better detect smaller differences in vancomycin resistance levels. All MM66 FARS mutants demonstrated increased distances grown on the vancomycin gradients examined compared to MM66, which indicated that the *apt* mutations altered vancomycin susceptibility levels. We conclude that 2-fluoroadenine-selected *apt* mutations can contribute to reduced vancomycin susceptibility in MM66 FARS mutants. 

Compared to MM66, the decrease in autolysis, increased adenine susceptibility and increase in vancomycin resistance exhibited by the MM66-FARS mutants, were not as great as that observed when we compared the MM66 VISA and MM66 (this study and [15]). This is likely due to the lack of the *walK* and/or *ssaA6* mutation in the MM66-FARS mutants. In addition, both VISA mutants which also harbored an *apt* mutation, demonstrated increased susceptibility to Congo red. This is in contrast to what was observed in the MM66 FARS mutants which demonstrated increased resistance to this dye. We propose that the additional mutations and altered cell wall physiology in the VISA mutants masked the effects of the *apt* mutation and supported the enhanced Congo red susceptibility observed.

Our results demonstrated that *sigB* and/or *sigB* operon genes are upregulated in the MM66 VISA strains. Since *sigB* is required for the full expression of vancomycin intermediate-levels in VISA strains [38,59,60], this finding was not unexpected. Having a primed general stress response might allow the MM66 VISA cell greater control over genes required for survival in the presence of vancomycin.

A large percentage of downregulated genes shared by both MM66 VISA investigated were represented by genes encoding virulence factors. In addition, our results demonstrate an increased in coagulation time and reduced hemolytic activity in the MM666 VISA mutants compared to the parent strain. These findings are in line with reports of downregulated virulence genes and reduced virulence factor production by other VISA strains [55].

Our transcriptional profiling data demonstrated the altered expression of a number of genes involved with the acquisition and biosynthesis of osmoprotectants in the MM66 VISA mutants. This finding in turn led us to investigate the growth of the MM66 VISA mutants in the presence of salt. While MM66 and the MM66 VISA mutants demonstrated similar growth curves in unamended media, the MM66 VISA mutants did not grow as well as parent strain MM66 in the presence of salt. Salt can cause the contraction of Gram-positive cell walls [61] and causes *S. aureus* to produce larger cells [62]. It is possible that salt affects the unique peptidoglycan metabolism/structural alterations in the MM66 VISA [3] in such a way that inhibits their growth greater than MM66. Salt addition has been shown to affect susceptibility to cell wall active antibiotics, for instance it is well know that salt addition positively affects the expression of resistance to cell wall active β-lactams by MRSA [63,64].

## 4. Materials and Methods

### 4.1. Bacterial Strains, Culture Conditions, and Chemicals

hVISA parent strain MM66 and MM66 VISA mutant strains MM66-3 and MM66-4 were utilized for this study [15]. All bacteria were cultured in Luria Bertani broth (LB) (Difco, Detroit, MI, USA) with shaking (200 rpm, 37 °C) or on LB agar (LBA) as required. Working stock LBA cultures were kept at 4 °C and all strains were stored in LB containing 20% glycerol at −80 °C. All overnight cultures were initiated with single colonies and then allowed to grow at 37 °C overnight. Growth experiments were performed in LB and in LB containing 2 M NaCl or 2 M KCl or 5 mM adenine or 5 mM 2-fluoroadenine. To make the LB plus salt media, NaCl or KCl crystals were added directly to the liquid media before autoclaving. Adenine and 2-fluoroadenine stocks were made up in DMSO and then filter sterilized before adding to liquid media. For growth experiments, triplicates of broth cultures were initiated with an overnight culture to reach an initial OD_580nm_ of 0.04 and the OD_580nm_ of each culture was recorded over the incubation time. Unless otherwise noted, chemicals were obtained from Sigma Chemical Co. (St. Louis, MO, USA).

### 4.2. DNA and RNA Purification and cDNA Synthesis

*S. aureus* chromosomal DNA was extracted by the DNA spooling method as described previously [65]. RNA for quantitative real-time PCR (qRT-PCR) and microarray analysis was isolated from mid-exponential phase cultures (OD_580nm_ = 0.7) using a bead mill homogenization procedure as previously described [66] following pretreatment of cell pellets with RNA protect (Qiagen Inc., Venlo, The Netherlands). cDNAs were synthesized from DNA*free*-treated (Ambion, Austin, TX, USA) RNA using Moloney murine leukemia virus SuperScript III reverse transcriptase (Invitrogen) as previously described [66].

### 4.3. Comparative Genome Sequencing

CGS services provided by Roche NimbleGen Inc. (Madison, WI, USA) were utilized for genomic mutation mapping to compare parent strain MM66 to VISA MM66-3. The *S. aureus* COL genome (Genbank accession: NC_002951 and NC_006629) tiling arrays were used to hybridize the test and the reference genomic DNAs, and single nucleotide polymorphisms in each strain were identified based on previously defined criteria [67]. All mutations identified by CGS (Roche NimbleGen) in MM66-3 were confirmed by sequencing PCR amplicons (Elim Biopharmaceuticals, Inc; Hayward, CA, USA) generated with primers described in Table S4. Sequencing reactions were performed with BigDye terminator chemistry and sequencing was done using ABI 3100 Genetic Analyzer (Applied Biosystem, Foster City, CA, USA). Mutations were detected with DNASTAR SeqMan Pro, (Version 9.1.0 (109), 2011). All genome sequence discrepancies detected by CGS in MM66-3 and MM66-4 were compared to nine *S. aureus* genomes: COL (laboratory MRSA), strain NCTC8325 (laboratory methicillin-susceptible *S. aureus* (MSSA), RF122 (bovine mastitis isolate), MW2 (community-acquired MRSA), MSSA476 (community-acquired MSSA), N315 (MRSA related to strain Mu50), USA300-FPR3757 (community-acquired MRSA), MRSA252 (epidemic MRSA), and Mu50 (MRSA-VISA). This was completed to help determine which mutations were unique to MM66-3 and MM66-4.

### 4.4. S. aureus DNA Microarray and Quantitative Real-Time PCR Analyses

For microarray analysis, cDNA samples prepared from MM66, MM66-3 and MM66-4 were labeled with Cy3 or Cy5 post-labeling reactive dye following the manufacturer’s suggestions (Amersham Biosciences, Piscataway, NJ, USA). Microarray experiments were performed in duplicate, and fluorophore dyes were swapped to produce dual cDNA samples to minimize dye bias for each strain cDNA preparation analyzed. *S. aureus* DNA microarrays version 4 were used for hybridization and array analysis. Hybridized arrays were scanned with a GenePix 4000B Microarray Scanner (Axon Instruments, Union City, CA, USA) and array TIFF images were analyzed using TIGR Spotfinder followed by data normalization with the LOWESS algorithm using TIGR-MIDAS. Genes that demonstrated ≥ 2.0-fold upregulation or downregulation were considered significant. The iCycler iQ Real-Time PCR Detection System (Bio-Rad Laboratories, Hercules, CA, USA) and iQ SYBR Green Supermix (Bio-Rad) were utilized for qRT-PCR of control and test cDNAs. The expression level of each sample was normalized using 16S rDNA as an internal control and expression ratios were determined using the 2^−∆∆Ct^ method as previously described [66]. All primers utilized for qRT-PCR are described in Table S4.

### 4.5. Isolation and Characterization of MM66 FARS Mutants

Aliquots (100 µL) of overnight cultures were spread on to LB agar containing 5 mM 2-fluoroadenine and incubated at 37 °C for 24 h. Colonies appearing on 2-fluoroadenine containing plates were then selected as suspected FARS-susceptibility mutants. MICs for 2-fluoroadenine were determined with LB overnight cultures, which were initially diluted to an OD_580nm_ of 0.01. A ten-microliter aliquot of each diluted overnight culture was then spotted onto LBA plates containing increasing concentrations of 2-fluoroadenine (0 and 4 to 12.0 mM at 0.5 mM increments). The spots were then allowed to dry, and plates were incubated at 37 °C for 24 h. The MIC was the lowest 2-fluoroadenine concentration at which there was no visible growth. A problem we encountered was that after 7 mM 2-fluoroadenine, it became clear that the drug was precipitating in the LBA causing the agar to take on a cloudy appearance, and therefore the final MICs could not be determined for the MM66 FARS mutants. In order to sequence and detect mutations in the *apt* genes of the MM66 FARS mutants a primer set (apt100U-F and apt100D-R) was designed that would amplify 100 bp upstream and downstream of the *apt* gene (Table S4). Sequencing of the *apt* amplicons and *apt* mutation detection was carried out as described above (Section 4.3). The determination of relative Congo red susceptibilities was carried out according to previous published protocols [12]. Vancomycin-resistance population and vancomycin gradient plate analyses were carried out as described previously [68,69].

### 4.6. Triton-X-100-Induced Whole Cell Autolysis, Coagulase Time and Hemolysis

Triton-X-100-stimulated whole cell autolysis assays were performed following the protocol of Gustafson et al. [70]. For coagulase assays, strains were grown in LB and the OD_580nm_ was adjusted to 1.0. Then, 50 µL of the OD-adjusted cultures were added to 500 µL of rehydrated coagulase plasma (BD BBL^TM^; Becton, Dickinson and Company, Sparks, MD, USA) and the time (min) to complete coagulation for all strains was then recorded. For hemolysis assays, overnight cultures were initially adjusted to an OD_580_ of 0.1. The cultures were then serially diluted in sterile LB and 100 µL aliquots of the 10^−6^ and 10^−7^ dilutions were spread onto the surface of 5% defibrinated sheep blood agar plates (Quad Five, Ryegate, MT, USA). The plates were then incubated at 37 °C for 24 h and then at 4 °C for 2 h. The sizes (mm) of the hemolysis zones around three isolated colonies of each strain investigated were then measured.

## Figures and Tables

**Table 1:** Confirmed mutations detected by comparative genome sequencing.

Strain		SACOL Loci ^a^	Gene	Function	SNP Position ^a^	Amino Acid Change
**MM66-3**						
	*Intergenic*					
		SACOL1918		IS1181 transposase	T^1974334^→A^1974334^	
		SACOL1919		Fur family transcriptional regulator		
		SACOL2608		Hypothetical protein	T^2667188^ → C^2667188^	
		SACOL2609		Hypothetical protein		
	*Intragenic*					
		SACOL1690	*apt*	Adenine phosphoribosyltransferase	C^1721075^ → T^1721075^	A^57^ → V^57^
		SACOL1576	*ssaA6*	CHAP domain containing protein	A^1610400^ → C^1610400^	H^297^ → G^297^
					T^1610401^ → C^1610401^	
					G^1610402^ → C^1610402^	
		SACOL1836		Hypothetical protein	T^1891424^ → C^1891424^	I^7^ →V^7^
		SACOL2639	*cysJ*	Sulfite reductase flavoprotein subunit	G^2699218^ → A^2699218^	P^458^ →S^458^

^a^ Based on loci numbers and nucleotide positions in NCBI Genbank database COL reference genome (discontinued in NCBI).

**Table 2:** Select transcriptionally altered genes in MM66 VISA mutants (TABLE MUST BE REPLACED).

SACOL Locus ^a^	Gene	Function	Fold Change in Gene Expression	
			MM66-3 (RT-qPCR)	MM66-4 (RT-qPCR)
Upregulated genes				
SACOL0136	*cap5A*	Capsular polysaccharide biosynthesis	2.9	4
SACOL0137	*cap5B*	Capsular polysaccharide biosynthesis	ND	2.7
SACOL0660	*adhP*	Alcohol dehydrogenase	2.1 (1.2)	2.3 (3.9)
SACOL0781	*opuAC*	Osmoprotectant ABC transporter component	ND	2.3
SACOL0856	*clfA*	Clumping factor A	2.1	ND
SACOL1078	*purL*	Phosphoribosylformylglycinamidine synthase II	2.2	2.2
SACOL1079	*purF*	Amidophosphoribosyltransferase	4.2	3.7
SACOL1080	*purM*	Phosphoribosylaminoimidazole synthetase	4.5 (4.5)	4.0 (3.3)
SACOL1081	*purN*	Phosphoribosylglycinamide formyltransferase	3.9	3.8
SACOL1082	*purH*	Phosphoribosylaminoimidazolecarboxamide	2.1	2.1
SACOL1083	*purD*	Phosphoribosylamine-glycine ligase	2.9	2.8
SACOL1215	*pyrAB*	Carbamoyl-phosphate synthase large subunit	3.1	2.5
SACOL1216	*pyrF*	Orotidine 5-phosphate decarboxylase	2.3	2.3
SACOL1217	*pyrE*	Orotate phosphoribosyltransferase	3.1	2.8
SACOL1328	*glnR*	Glutamine synthetase repressor	2	3.6
SACOL1450	*arlS*	Sensor histidine kinase	2	2.1
SACOL1451	*arlR*	DNA-binding response regulator	2.2	3.2
SACOL2054	*sigB*	RNA polymerase sigma factor *sigB*	ND (2.0)	2.3 (1.4)
SACOL2055	*rsbW*	Anti-sigma B factor	ND	2.9
SACOL2056	*rsbV*	Anti-anti-sigma factor	ND	2.2
SACOL2088	*sceD*	*sceD* autolysin	2.7	3.7
SACOL2092	*murA*	UDP-N-acetylglucosamine 1-carboxyvinyltransferase	ND	2.7
SACOL2176	*opuD1*	BCCT family osmoprotectant transporter	2.2	2.3
SACOL2181	*lacE*	PTS system, lactose-specific IIBC components	12.7	6.1
SACOL2182	*lacF*	PTS system, lactose-specific IIA component	10.8 (4.0)	9.0 (3.2)
SACOL2187	*lacA1*	Hypothetical protein	14	9.4
SACOL2671	*secA*	*secA* preprotein translocase subunit	2.7	3.1
Downregulated genes				
SACOL0209	*coa*	Staphylocoagulase precursor	−3.0 (−1.1)	−3.3 (−2.1)
SACOL0478	*ssl11*	Superantigen-like protein	ND	−2.1
SACOL0507	*aaa*	Multifunctional autolysin	ND	−3.2
SACOL0723	*aaa1*	*aaa* homolog (38 % identity)	ND	−2.9
SACOL1062	*atl*	Bifunctional autolysin	ND	−2
SACOL1164		Fibrinogen binding-related protein	−3.9	−3.9
SACOL1169		Fibrinogen-binding protein precursor-related protein	−2.5	−3.4
SACOL1173	*hly*	Alpha-hemolysin precursor	−2.4 (−1.9)	−5.0 (−2.6)
SACOL2291	*ssaA1*	Secretory antigen precursor *ssaA*	ND	−2.2
SACOL2295	*ssaA3*	*ssaA* homolog	ND	−2.2
SACOL2418	*sbi*	IgG-binding protein	−2.3 (−1.2)	−3.3 (−3.0)
SACOL2421	*hlgC*	Gamma-hemolysin, component C	ND	−2.5
SACOL2422	*hlgB*	Gamma hemolysin, component B	ND	−2.2
SACOL2581	*ssaA2*	*ssaA* homolog	ND	−3.1
SACOL2627	*betA*	Choline dehydrogenase	ND	−7.4
SACOL2628	*betB*	Glycine betaine aldehyde dehydrogenase	ND (−2.2)	−9.2 (−7.0)
SACOL2632	*opuD2*	BCCT family osmoprotectant transporter	ND	−4.6

^a^ Gene ID correspond to strain COL genome. Abbreviations: ND, transcriptional alteration not detected.

**Table 3:** Selection of MM66 FARS mutants.

Strain	Parent Strain	2-FA Selection Conc. (mM)	2-FA MIC (mM)	References
MM66			5	[15]
MM66-FA-1	MM66	5	>7	This study
MM66-FA-2	MM66	5	>7	This study
MM66-FA-3	MM66	5	>7	This study
MM66-FA-4	MM66	5	>7	This study
MM66-FA-5	MM66	5	>7	This study
MM66-FA-6	MM66	5	>7	This study

**Table 4:** *apt* mutations in MM66 FARS mutants.

2-FA^RS^ Mutant(s)	*apt* Mutation *	Effect of Mutation on Apt
MM66-FA-1	Contiguous 45 bp deletion between A^1720937^—A^1720891^	M^103^ →I^103^ and H^104^—D^118^ (HKDAIKPGQRVLITD) internal deletion
MM66-FA-2	Contiguous 45 bp deletion between A^1720937^—A^1720891^	M^103^ →I^103^ and H^104^—D^118^ (HKDAIKPGQRVLITD) internal deletion
MM66-FA-3	C insertion after GCACC^1721018^	Frameshift after P^76^
MM66-FA-4	Noncontiguous 15 bp deletion between C^1721039^—A^1721020^	M^70^—F^74^ (MGIGF) internal deletion
MM66-FA-5	ATGGG insertion after TGGGG^1701031^	Frameshift after G^71^
MM66-FA-6	T^1720975^ →A^1720975^	Y^90^ →stop codon (TAA)

* Based on nucleotide positions in NC_002951.

**Table 5:** The distances (mm ± SD, *n* = 3) grown by MM66 and MM66 FARS mutants on vancomycin gradients.

Strain	Vancomycin Gradients	
	0–2 mg/L	0–2.5 mg/L
MM66	26 ± 2.00	17 ± 1.00
MM66-FA-1	47 ± 0.58 *	33 ± 2.52 *
MM66-FA-2	48 ± 3.05 *	33 ± 2.65 *
MM66-FA-3	49 ± 3.06 *	36 ± 1.00 *
MM66-FA-4	52 ± 0.58 *	37 ± 0.00 *
MM66-FA-5	55 ± 2.52 *	38 ± 1.00 *
MM66-FA-6	53 ± 1.53 *	36 ± 0.58 *

* *p*-value ≤ 0.05 in comparison to MM66.

## Data Availability

All microarray data can be found in the NCBI’s Gene Expression Omnibus and is accessible through GEO Series accession number GSE16479 (http://www.ncbi.nlm.nih.gov/geo/query/acc.cgi?acc=GSE16479, accessed on 31 January 2021).

## Supplementary Materials

The following are available online at https://www.mdpi.com/2079-6382/10/5/583/s1, Table S1: Genes altered ≥2-fold in MM66-3 compared to MM66; Table S2: Genes altered ≥2-fold in MM66-4 compared to MM66; Table S3: Genes altered ≥2-fold in both MM66-3 and MM66-4 compared to MM66; Table S4: All primers used in study.
